# Forging Links between Human Mental Retardation–Associated CNVs and Mouse Gene Knockout Models

**DOI:** 10.1371/journal.pgen.1000531

**Published:** 2009-06-26

**Authors:** Caleb Webber, Jayne Y. Hehir-Kwa, Duc-Quang Nguyen, Bert B. A. de Vries, Joris A. Veltman, Chris P. Ponting

**Affiliations:** 1 MRC Functional Genomics Unit, Department of Physiology, Anatomy and Genetics, University of Oxford, Oxford, United Kingdom; 2 Department of Human Genetics, Nijmegen Centre for Molecular Life Sciences, Radboud University Nijmegen Medical Centre, Nijmegen, The Netherlands; Wellcome Trust Sanger Institute, United Kingdom

## Abstract

Rare copy number variants (CNVs) are frequently associated with common neurological disorders such as mental retardation (MR; learning disability), autism, and schizophrenia. CNV screening in clinical practice is limited because pathological CNVs cannot be distinguished routinely from benign CNVs, and because genes underlying patients' phenotypes remain largely unknown. Here, we present a novel, statistically robust approach that forges links between 148 MR–associated CNVs and phenotypes from ∼5,000 mouse gene knockout experiments. These CNVs were found to be significantly enriched in two classes of genes, those whose mouse orthologues, when disrupted, result in either abnormal axon or dopaminergic neuron morphologies. Additional enrichments highlighted correspondences between relevant mouse phenotypes and secondary presentations such as brain abnormality, cleft palate, and seizures. The strength of these phenotype enrichments (>100% increases) greatly exceeded molecular annotations (<30% increases) and allowed the identification of 78 genes that may contribute to MR and associated phenotypes. This study is the first to demonstrate how the power of mouse knockout data can be systematically exploited to better understand genetically heterogeneous neurological disorders.

## Author Summary

Mental retardation (MR; also known as learning disability) affects 1%–3% of people and is often associated with the presence of genomic copy number variations (CNVs) such as deletions and duplications. Most of these CNVs are rare and they often involve tens, sometimes hundreds, of genes. Pinpointing exactly which particular gene or genes are responsible for MR in an individual patient is therefore challenging and limits diagnostic applications. In this study, the functions of genes present within a large collection of MR–associated CNVs were investigated by comparing them to data from large-scale mouse knock-out experiments. We found that MR–associated CNVs contain greater than expected numbers of genes that give specific nervous system phenotypes when disrupted in the mouse. Not only does this study confirm that CNVs frequently cause MR, but it narrows down the list of genes whose changes lead to this disorder from thousands to several dozen. This reduced list of genes brings wide-spread genetic testing for MR one step closer. It also provides a better understanding of the biology behind MR that could, eventually, yield medical treatments.

## Introduction

Mental retardation (MR) is defined as an overall intelligence quotient lower than 70, and is associated with functional deficits in adaptive behaviour, such as daily-living skills, social skills and communication. This disorder affects 1%–3% of the population and results from extraordinarily heterogeneous environmental and genetic causes [1]. Genetic changes underlying MR are still poorly resolved, especially for the autosomes that provide the largest contribution to disease aetiology [2]. Microscopically visible chromosomal rearrangements detected by routine chromosome analysis are the cause for MR in ∼5%–10% of patients [3]. Such rearrangements represent gains or losses of more than 5–10 Mb of DNA and affect many genes thereby almost inevitably leading to developmental abnormalities during embryogenesis. The most common effect of these variants is cognitive impairment, but they can also be frequently associated with other abnormalities such as heart defects, seizures and dysmorphic features [4].

Many recent genomic microarray studies have indicated that smaller, submicroscopic rearrangements, such as copy number variations (CNVs), frequently underlie MR (Table S1). However, CNVs, defined as DNA deletions or duplications greater than 1 Kb [5], are also widespread in the general population which considerably hinders the clinical interpretation of patients' CNVs [6]. Until now, most clinical CNV studies have focused on the identification of rare *de novo* CNVs [7]–[9], as the rate of *de novo* large (>50 kb) CNVs in the general population is comparatively low [10],[11]. Nevertheless, discriminating between benign and pathogenic CNVs solely on the basis of size and lack of inheritance is crude and provides no insights into how CNVs exert their phenotypic effects.

Fortunately, the genomics era has amassed a wealth of data that have long promised to associate the disruption of a particular molecular function or cellular pathway with clinical observations; in short, to forge links between genotype and disease phenotype. These genomic data include behavioural, physiological and anatomical examinations following the disruption of more than 5000 individual mouse genes [12]–[14]. These mouse phenotypic measurements more closely resemble observations from human clinical examination than any other systematic genome-wide data source. They might be especially relevant to human gene deletion variants, which represent a large majority among the rare disease-associated CNVs considered here (Table 1 and Table S2). Available genomic data also include functional annotations such as from the Gene Ontology resource [15], tissue expression levels [16] and carefully curated pathway data such as the Kyoto Encyclopedia of Genes and Genomes (KEGG) [17].

**Table 1:** Genomic extent and NCBI gene content for MR–associated and benign CNVs.

	CNVR number (median size)	CNV number (median size)	Gene Count	MR CNV genes also contained within benign CNVs	MR CNV genes not contained within benign CNVs	Genome covered (Mb)	Gene density/Mb
**All MR**	112 (2.76 Mb)	148 (2.74 Mb)	4,009	703	3,397	440.1	9.1
**Gain MR**	32 (1.90 Mb)	37 (2.55 Mb)	1,189	283	907	92.9	12.8
**Loss MR**	85 (3.04 Mb)	111 (2.85 Mb)	3,159	449	2,711	367.8	8.6
**Benign**	1,388 (0.17 Mb)	26,472 (0.21 Mb)	4,576	N/A	N/A	429.0	10.7

Our approach was to test the null hypothesis that genes present in MR–associated CNVs randomly sample all human genes. In particular, are they a random sample of genes (*i*) that, when disrupted in mice, result in particular phenotypes, or (*ii*) that are predominantly expressed in the human brain, or (*iii*) that participate in specific human disease pathways? To ensure that we correctly account for the application of multiple tests, we have controlled the false discovery rate (FDR) [18] such that there is only a small 5% likelihood that any annotation term has been identified as over-represented in our tests simply by chance. Only if any particular set of genes present within MR–associated CNVs form a significantly (FDR<5%) non-random sample can we be truly justified in predicting single genes, among the dozens commonly overlapped by such CNVs, as contributing to MR disease aetiology. In this study, we show both significant and substantial enrichments in phenotypic annotations whose power in predicting pathoetiology greatly exceeds that of molecular annotations.

## Results

For this study, 148 MR–associated rare CNVs collated from a variety of sources (Table S1) were merged to obtain a set of 112 distinct non-overlapping CNV regions (CNVRs) and partitioned according to the direction of copy number change (*Gain* or *Loss*). We also collated a control set of 26,472 benign CNVs (1,388 CNVRs) from previous publications (see Materials and Methods). MR–associated CNVs are most obviously distinguished from benign CNVs by their large sizes and by their larger numbers of copy number losses (*n* = 111, 75%) relative to gains (*n* = 37, 25%) (Table 1). These differences remained even when comparing benign and MR CNVs detected by the same platform (tiling resolution 32 k BAC arrays): the median size of 40 MR CNVs is approximately twice that of benign CNVs (1.6 Mb *versus* 0.85 Mb) while 58.6% of benign CNVs on this platform are losses. This increased bias towards loss CNVs would be expected if the MR phenotypes considered here result either from haploinsufficiency or from recessive deleterious mutations being revealed in the remaining haplotype. There is only a small difference (17.6%) between the average gene densities of MR–associated and benign CNVs (Table 1). Consequently, we need to look to gene function, rather than gene numbers, when attempting to differentiate disease-associated from benign CNVs.

### Nervous system phenotypes and expression

**Figure 1:**
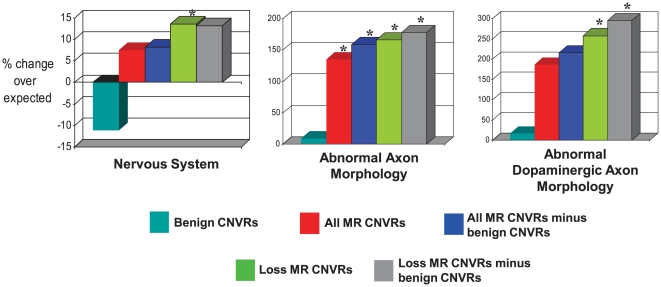
Enrichments of MGI phenotype terms among genes overlapped by MR–associated CNVRs. One phenotypic category (Nervous System) and two specific nervous system phenotypes (Abnormal Axon Morphology and Abnormal Dopaminergic Neuron Morphology) are significantly over-represented in genes overlapped by *All* or *Loss*-only MR–associated CNVRs. The phenotypes result from the disruption of mouse genes that have been mapped to their unique human orthologue. MR CNVR sets denoted “minus benign CNVs” have had genes removed that are also overlapped by benign CNVRs when matched on the direction of copy number change (i.e. *Gain* or *Loss*). Columns marked with an asterisk (“*”) are significantly enriched (FDR<5%).

We first tested whether MR–associated CNVR genes were enriched in 33 major categories of mouse phenotypes (see Materials and Methods). Although for *All* MR–associated CNVRs none of these terms was significant, the set of *Loss* MR–associated CNVRs showed a strong and significant enrichment in genes whose knockouts in mice produced a nervous system phenotype (+13.6%, or 1.14-fold, enrichment, *p* = 3×10^−3^, FDR<5%; Figure 1). An enrichment of genes associated with nervous system phenotypes was not observed within the *Gain* CNVRs (+0.2%).

Given the significant enrichment within the *Loss* set, we then tested this set against each of 147 finer-scale mouse nervous system phenotypes. Two of these terms were significantly enriched (FDR<5%): abnormal axon morphology (*obs* = 19, *exp* = 7.1, +170% enrichment, *p* = 3×10^−5^), and abnormal dopaminergic neuron morphology (*obs* = 9, *exp* = 2.5, +260% enrichment, *p* = 3×10^−4^) (Figure 1). Both of these mouse neural phenotypes are relevant to human MR phenotypes owing to these mouse phenotype's abnormalities in neuronal and cerebral cortex morphologies (see Discussion). Within *Gain* CNVRs, we observe a non-significant enrichment of genes associated with abnormal axon morphology (*obs* = 6, *exp* = 2.7, +120% enrichment, *p* = 5×10^−2^) but a non-significant depletion of genes associated with abnormal dopaminergic neuron morphology (*obs* = 0, *exp* = 0.95, −100% deficit, *p* = 0.38).

**Figure 2:**
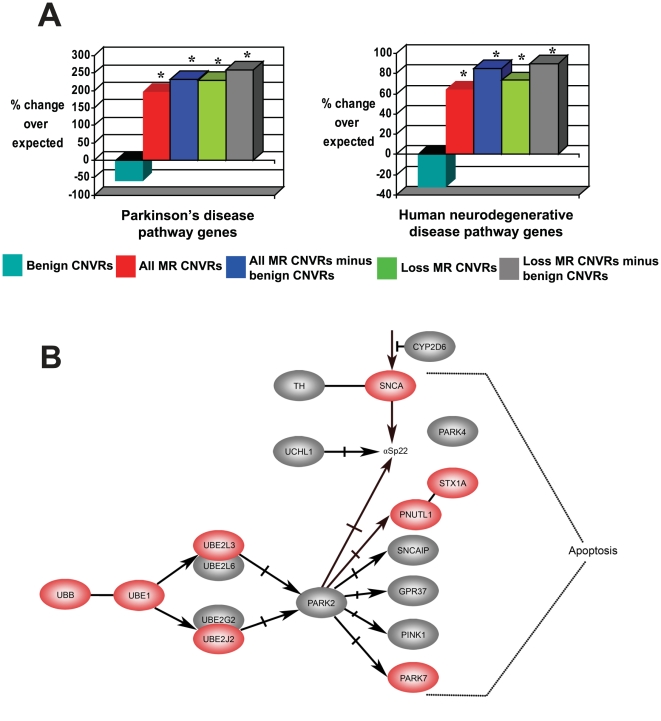
Human gene enrichments corroborate mouse phenotypic enrichments. (A) Enrichments of genes involved in Parkinson's disease or human neurodegenerative disease pathways that are overlapped by MR–associated CNVRs. These genes are described by KEGG as belonging to the Parkinson's disease pathway (HSA05020) or belonging to any of six neurodegenerative pathways (namely, HSA05010, HSA05020, HSA05030, HSA05040, HSA05050, and HSA05060). MR–associated CNVR sets denoted “minus benign CNVs” have had genes removed that are also overlapped by benign CNVRs showing the same direction of copy number change (i.e. *Gain* or *Loss*) as its overlapping MR–associated CNVR. Columns marked with an asterisk (“*”) are significantly enriched (FDR<5%). (B) All genes contained in the KEGG Parkinson's disease pathway (HSA05020). Of the 18 genes in this pathway, 8 (highlighted in red) are involved in a rare *de novo* CNV from at least one or more patients. The remaining genes (depicted in grey) lie outside of the 148 MR CNVs that we considered.

The neurological phenotypes of MR patients suggested that MR–associated CNVs might contain an unusually high density of genes that, when mutated, are involved in human neurological disease. Considering those genes classified by KEGG to be involved in 6 neurodegenerative pathways, we indeed found MR–associated CNVRs to be significantly enriched in genes involved in the Parkinson's disease pathway (*obs* = 8, *exp* = 2.7, +196% enrichment, *p* = 3×10^−3^, FDR<5%; Figure 2). While enrichments of this pathway's genes were observed both for *Loss* CNVRs (*obs* = 7, *exp* = 2.1, +230% enrichment, *p* = 3×10^−3^, FDR<5%) and for *Gain* CNVRs (*obs* = 2, *exp* = 0.8, +151% enrichment, *p* = 0.19), significance was reached only for *Loss* CNVRs. As Parkinson's disease is a condition characterized by the degeneration and dysfunction of dopaminergic neurons [19], these enrichments corroborate our finding that orthologues of genes whose disruption in mouse gives rise to abnormal dopaminergic neuron morphology are enriched in MR–associated CNVRs (see above).

**Figure 3:**
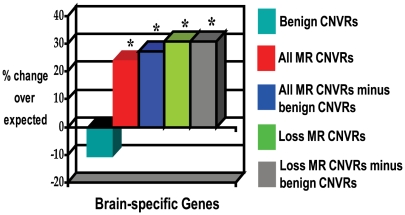
Enrichment of genes, overlapped by MR–associated CNVRs, that are expressed highly in the brain relative to other-tissue (*brain-specific* genes). Such genes are defined as those whose level of expression in the brain exceeds 4 times the median expression level in all other tissues (see Materials and Methods). MR–associated CNVR sets denoted “minus benign CNVs” have had genes removed that are also overlapped by benign CNVRs showing the same direction of copy number change (i.e. *Gain* or *Loss*) as its overlapping MR–associated CNVR. Columns marked with an asterisk (“*”) are significantly enriched (FDR<5%).

The allelic changes underlying MR phenotypes might also be expected to preferentially involve ‘brain-specific’ genes, those that are highly expressed in the human brain relative to other human tissues. Indeed, *All* MR–associated CNVRs were significantly enriched in brain-specific genes (+24% enrichment, *p* = 1×10^−2^; Figure 3), specifically for *Loss* (+31% enrichment, *p* = 8×10^−3^) but not for *Gain* CNVs (+4% enrichment, *p* = 0.45). The significant enrichments observed when testing mouse phenotypes are thus corroborated by enrichments in human gene expression.

### Distinction from benign CNVs

These findings would have little or no predictive potential if apparently ‘benign’ CNVs (those present in the general human population) also exhibit such biases. However, in contrast to the above results, benign CNVs show no significant enrichments of (*i*) genes that are highly-expressed in the brain (−11% deficit, *p* = 0.2; Figure 3), (*ii*) genes present in neurodegenerative disease pathways (−32% deficit, *p* = 0.1; Figure 2), or (*iii*) genes with nervous system phenotypes when disrupted in mice (−11% deficit, *p* = 0.01; Figure 1). Instead, benign CNV genes show significant tendencies to encode proteins with roles in immunity and host defense [20],[21]. Each of these three features thus may be exploited to distinguish MR–associated CNVR genes from benign CNVR genes.

MR–associated and benign CNVs show no significant tendency to overlap (*p* = 0.1). Nevertheless, by excluding all genes in MR–associated CNVs whose gain/loss-matched copy number change is also seen in benign CNVs we enhanced the discrimination of genes whose copy number change is predicted to contribute to MR aetiology. This was specifically the case for mouse fine-scale nervous system phenotypes and human neurodegenerative disease pathways (Figure 1 and Figure 2). Moreover, after excluding benign CNV-overlapped genes, not only Parkinson's disease pathway genes, but genes from 5 other neurodegenerative disease pathways (namely, Alzheimer's disease, Amyotrophic Lateral Sclerosis, Huntington's disease, Dentatorubropallidoluysian atrophy and Prion Diseases) when considered together, became significantly enriched (+60% enrichment; *p* = 0.02) in this analysis. These results would be explained if MR-causative alleles segregate more with sequence that is copy number variable in MR individuals than with CNVs observed in the general population.

### Additional clinical features

**Figure 4:**
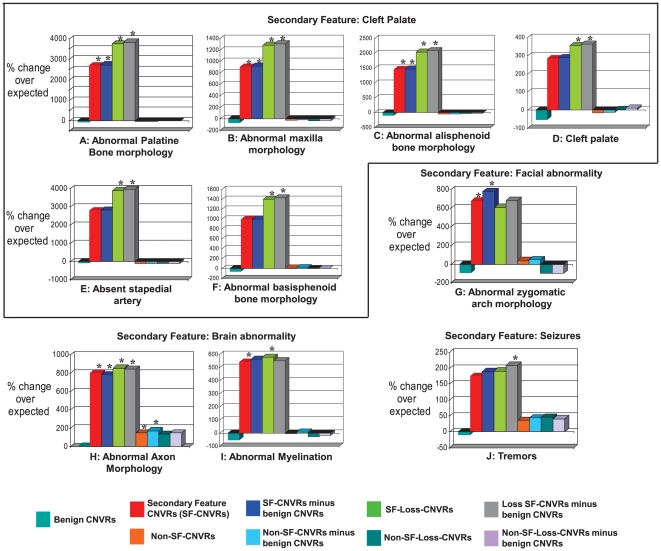
Enrichments of MGI phenotype terms for genes overlapping secondary clinical feature-grouped CNVRs. 5 secondary feature-grouped CNVs revealed between 1–6 significantly enriched phenotypic terms (Cleft Palate, panels (A) to (F); Facial abnormality, panel (G); Brain Abnormality, panels (H) and (I); Seizures, panel (J)). These MGI terms are significantly over-represented in genes overlapped by *All* or *Loss*-only secondary feature-grouped CNVRs (see main text). The phenotypes result from the disruption of mouse genes that have been mapped to their unique human orthologue. MR CNVR sets denoted “minus benign CNVs” have had genes removed that are also overlapped by benign CNVRs showing the same direction of copy number change (i.e. *Gain* or *Loss*) as its overlapping MR–associated CNVR. Columns marked with an asterisk (“*”) are significantly enriched (FDR<5%).

We considered whether our method could identify significant associations between mouse and human patient phenotypes other than MR. We investigated 7 clinical features that were present in our patient population in addition to the MR phenotype, namely brain-, cleft palate-, eye-, facial-, heart- or urogenital- abnormalities and seizures (see Materials and Methods). We tested whether CNVs from individuals with these specific clinical features were significantly enriched in genes associated with phenotypically-relevant mouse phenotypes. In order to limit the large number of statistical tests that could be performed we matched mouse phenotype categories (each containing between 129 and 220 terms) to each of the 7 clinical features based on clinical experience (see Materials and Methods) before performing the association tests. We found that 4 of the 7 additional clinical features were significantly associated (FDR<5%) with between 1 and 6 mouse phenotypic terms (Figure 4). For example, the CNVRs of the 8 MR patients presenting with cleft palate were significantly enriched with genes whose mouse orthologues, when disrupted, also exhibited cleft palate (Figure 4). Importantly, no significant associations were observed between CNVs from humans without a particular clinical feature apart from MR and any mouse phenotype category matched to patients with that clinical feature, with the notable exception of ‘abnormal axon morphology’ that thus appears to be a term of broad relevance to the primary MR presentation (Figure 4). These findings demonstrate the relevance of mouse gene knockout observations to both the MR phenotype and associated phenotypes in patients.

### Predicting genetic etiology

The distinctions between MR–associated and benign CNVR genes, described above, allowed the identification of genes whose copy number change may contribute to MR and associated phenotypes. To identify such candidate genes, we could not exploit Gene Ontology annotations (Figure S1) or brain expression enrichments (Figure 3) as these enrichments provide insufficient discriminatory power (<30% increase over expected). Of the 4,009 genes present in the 148 MR–associated CNVs, 55 are annotated with either a mouse knockout phenotype (*n* = 29) and/or a neurodegenerative disease pathway (*n* = 29) that was significantly over-represented in MR–associated *Loss* CNVRs (Table 2). 50 of the MR–associated CNVs (33%) contain at least 1 of these 55 candidate genes. We calculate that our list represents a ∼120% increase of likely phenotype-contributing genes over the random expectation (see Materials and Methods). Similarly, 34 genes were identified as potential candidates for additional clinical features such as cleft palate, facial or brain abnormalities, or seizures, 23 of which were not associated with MR itself (Table 2). We note that whilst some of these candidate genes might have been prioritized from among the 4,009 CNVRs genes using *a priori* subjective expectations, our method is the first to generate a candidate gene set on the basis of objective and statistically sound criteria.

**Table 2:** Candidate genes for MR and associated clinical features.

MGI phenotype or KEGG pathway	*Gene in Loss MR CNVR*			*Gene in Gain MR CNVR*
- ***associated with Mental Retardation***				
Abnormal dopaminergic neuron morphology (MP0003243)	*EN1*	*LOC390992*	*SLC6A3*	
	*HES1*	*MAPK10*	*SNCA*	
	*KCNJ6*	*SLC18A2*	*SPP1*	
Abnormal axon morphology (MP0005404)	*APG5L*	*MAPT*	*PTPN13*	*LGI4*
	*ARSA*	*MBP*	*SCN1B*	*MAG*
	*CLCN6*	*MFN2*	*SIM1*	*SCN1B*
	*LEPR*	*NEFH*	*SNCA*	*SCYL1*
	*LGI4*	*NR2F1*	*TYROBP*	*TYROBP*
	*MAG*	*PLP1*		*ZIC5*
	*MAN2B1*	*PMP22*		
KEGG Neurodegenerative Pathway genes	*ALS2*	*HSPD1*	*RERE*	*APLP1*
	*APLP1*	*MAPT*	*SNCA* *	*BAD*
	*BACE2*	*NCOR1*	*SOD1*	*CLTCL1*
	*CAGLP*	*NEFH*	*SSR4*	*CREBBP*
	*CASP7*	*PARK7* *	*STX1A* *	*HADH2*
	*CASP8*	*PEN2*	*UBB* *	*PEN2*
	*CLTB*	*PNUTL1* *	*UBE2J2* *	*PNUTL1*
	*CLTCL1*	*RAC1*	*UBE2L3* *	*UBE1* *
	*HD*			
- ***associated with Brain Abnormality***				
Abnormal myelination (MP0000920)	*HPN*	*OLIG2*		*HPN*
	*LGI4*	*PLP1*		*LGI4*
	*MAG*	*TYROBP*		*MAG*
				*TYROBP*
Abnormal axon morphology (MP0005404)	*LGI4*	*PLP1*		*LGI4*
	*MAG*	*SCN1B*		*MAG*
	*MAPT*	*TYROBP*		*SCN1B*
	*NR2F1*			*TYROBP*
- ***associated with Cleft Palate***				
Abnormal basisphenoid bone morphology (MP0000106)	*DISP1*	*DLX2*		
	*DLX1*			
Cleft palate (MP0000111)	*DLX1*	*GAD1*		*CREBBP*
	*DLX2*	*LHX8*		
	*EDNRA*	*MN1*		
Abnormal maxilla morphology (MP0000455)	*DLX1*	*EDNRA*		
	*DLX2*	*GAD1*		
Abnormal alisphenoid bone morphology (MP0003235)	*DLX1*	*EDNRA*		
	*DLX2*			
Absent stapedial artery (MP0004666)	*DLX1*	*DLX2*		
Abnormal palatine bone morphology (MP0005249)	*DLX1*	*LHX8*		
	*DLX2*	*MN1*		
	*EDNRA*			
- ***associated with Facial Dysmorphism***				
Abnormal zygomatic arch morphology (MP0004469)	*ACVR1*	*TBX1*		*IDUA*
	*CHRD*	*ZMPSTE24*		*NFATC2*
	*IDUA*			*TBX1*
- ***associated with Seizures***				
Tremors (MP0000745)	*ATF2*	*KCNAB2*	*SELE*	
	*EN1*	*KCNJ6*	*SELP*	
	*ESPN*	*MAPT*	*SLC25A12*	
	*GLI2*	*SCN1A*	*ZMPSTE24*	
	*HD*			

These are present in MR–associated CNVRs and belong to any of three significantly enriched annotations; namely, mouse knockout phenotypes of abnormal dopaminergic neuron morphology or abnormal axon morphology (Figure 1), and KEGG neurodegenerative pathway genes (Figure 2). Neurodegenerative pathway genes within the Parkinson's disease pathway are marked with an asterisk (‘*’). The remaining genes lie within CNVs associated with the particular secondary clinical features and belong to significant enrichments identified as specific to those clinical feature.

## Discussion

If *de novo* MR–associated CNVs do not contribute to disease etiology their gene contents would not be expected to exhibit biases in gene function or expression. Instead, we demonstrate the first evidence for significant tendencies of MR–associated CNV genes to be brain-expressed, to belong to neurodegenerative pathways, and to present particular phenotypes when disrupted in mice, all of which validate the assumption that large *de novo* CNVs commonly underlie MR phenotypes. These results could not have been obtained without collating data from a number of sources. For example, essentially all (147 of 148) CNVs were required to obtain a significant enrichment of genes whose mouse orthologues' knockout produced a nervous system phenotype (Figure S2). It was only by harnessing the statistical power of a research community's large data set that this meta-analysis achieved significance of statistical associations (see Materials and Methods).

The significant signals seen in *Loss* CNVs, but not in *Gain* CNVs, imply that MR phenotypes commonly result from gene dosage sensitivity (haploinsufficency). However, we cannot discount that they may occur from the uncovering, by DNA loss, of rare recessive alleles. While we did not observe an enrichment within the *Gain* CNVRs of genes associated with abnormal dopaminergic neuron morphology or of genes that showed brain-specific expression, we did observe non-significant enrichments of genes associated with abnormal axon morphology and of Parkinson's disease pathway genes. Given that the *Gain* CNVRs overlap 38% of the number of genes overlapped by the *Loss* CNVRs (Table 1), it is plausible that these enrichments might reach significance as more *Gain* MR–associated CNVs are reported and analysed.

Our results are in contrast with previously-reported sporadic and familial cases of MR whose associated genes are enriched in both X-chromosome location and enzymatic function [22]. Nevertheless, this is explained by Wright's physiological theory of dominance: haplosufficient genes, such as those lying on the X chromosome, have an expected tendency to encode enzymes, whereas haploinsufficient genes, such as those expected to underlie our autosomal MR disorders, have an expected tendency to encode transcription regulatory genes [23]. Indeed, we do observe a significant enrichment of genes associated with transcriptional regulation within MR–associated CNVRs (Figure S1). In contrast to X-linked MR genes, of which approximately one quarter encode postsynaptic proteins [24], we observe a small and non-significant depletion (*p* = 0.39) of postsynaptic protein genes among our MR–associated CNVs.

None of the human CNVs recorded in this study represent homozygous losses. Thus it may initially appear problematic to compare human phenotypes directly with those from mice harbouring homozygous gene disruptions. Nevertheless, without sequence information confirming the genetic integrity of the surviving haplotype we cannot be certain that these human hemizygous loss CNVs do not contain independent disruptions of each allelic copy. To gain some insight into this issue we considered 21 of the 55 candidate genes that contribute to a significantly enriched mouse knock-out phenotype identified in our study (Table 2), and whose phenotype has been recorded in the MGI resource when in the hemizygous state. Of these 21, four (namely, *En1*, *Mn1*, *Plp1* and *Pmp22*) also exhibit the phenotype of interest when hemizygously disrupted [25]–[28]. Of the remaining 17 genes, all exhibit abnormal phenotypes, and thus are haploinsufficient, with the exceptions of *Mapt* and *Slc6a3* [29],[30]. Importantly, these mouse hemizygous phenotypes are often closely-related to the homozygous phenotypes, while some hemizygous phenotypes appear particularly relevant to the associated human phenotype. For example, *Scn1a* (which contributes to the tremors phenotypic enrichment we find to be associated with patients presenting with seizures) exhibits a seizures phenotype when in the hemizygous state in mice [31].

Does our analysis allow us to link particular mouse gene knockout phenotypes to human CNV phenotypes? Obviously, a direct comparison between mouse neural phenotypes and human MR phenotypes is hindered because the invasive procedures of brain biopsies in patients are unacceptable. Results from a limited number of post-mortem studies of MR patients suggest that abnormalities of dendritic spines are a general neuropathological feature of MR [32]. The mouse gene knockout phenotypes do provide a plausible explanation for the brain phenotypes observed in some patients as a consequence of the structural variation identified in their genomes. An example of this is the myelin-associated glycoprotein (*MAG*) gene that is deleted in one patient (case 123, Table S2) and duplicated in another (case 124), whilst the knockout of its orthologous gene in mice leads to both abnormal axon morphology and tremors phenotypes [33]. Underexpression of *MAG* in transfected Schwann cells is known to lead to hypomyelinisation [34]. Therefore, the delayed brain myelinisation observed in the patient with the *MAG* deletion could be caused by under-expression of *MAG* during brain development. By contrast, over-expression of *MAG* is known to lead to accelerated myelinisation [35]. Whether the macrocephaly in the patient with the *MAG* duplication is related to over-expression of *MAG* during brain development remains unknown.

Our enrichment analysis revealed 8 genes associated with cleft palate in humans, present in 6 different patients (cases 10, 13, 27, 48, 96, and 141). Seven of these genes were located in *Loss* CNVs on human chromosomes 1p31.1p31.3 (containing *LHX8*), 1q41q42.13 (*DISP1*), 2q24.3q31.1 (*DLX1*, *DLX2* and *GAD1*), 4q31.21q31.23 (*EDNRA*) and 22q12.1 (*MN1*), and one with a *Gain* CNV on human chromosome 16p13.2–p13.3 9 (*CREBBP*). Except for *DISP1*, all these genes have been associated with cleft palate in mouse models [26], [36]–[39], whereas only *LHX8* and *GAD1* have been associated with cleft palate disorders in humans [40],[41]. This strongly suggests that our approach revealed 6 novel orofacial cleft (OFC) candidate genes in humans. Strikingly, the hemizygous loss of five of these OFC candidate genes may also contribute to MR. Absence of both *Dlx1* and *Dlx2* in mice results in abnormal differentiation within the forebrain [36],[42]. Both genes also regulate *Arx*, a homeobox transcription factor required for the migration of interneurons, whose human equivalent *ARX*, when mutated, is associated with X-linked MR and epilepsy [43]. In addition, mutations and deletions of *CREBBP* causes the Rubinstein-Taybi syndrome which is characterized by MR [44]. *Ednra* is involved in cranial neural crest cell migration from the posterior midbrain and hindbrain to the arches [45]. *Lhx8* is required for the development of many cholinergic neurons in the mouse forebrain [46], whereas *GAD1*, which encodes the GABA-producing enzyme, may play a role in the development and plasticity of the central nervous system [39]. In conclusion, it appears that our approach identified a large number of interesting and plausible novel candidate genes for both MR and associated clinical phenotypes.

Mouse phenotype data have not previously been exploited in a systematic genome-wide analysis, and our results clearly show its utility in addressing a particularly difficult and contemporary challenge in the field of neurological genomic disorders. The functional biases we see for MR–associated CNV genes can now be exploited to prioritise genes for further investigation in MR individuals without large *de novo* CNVs (Table 2). We suggest that all human genes whose orthologues present specific phenotypes when disrupted in mice (Figure 1) deserve particular scrutiny for fine-scale insertion, deletion or point mutations contributing to MR. Mouse orthologue knockout data are available currently for only ∼25% of all human genes. More specifically, of the 4,009 genes overlapped by the MR–associated CNVs considered here, 830 (∼21%) have available phenotypic annotations. Thus, we would expect that many more candidate genes possessing these annotations will be discovered within MR–associated CNVs as further knockouts are generated. Furthermore, we consider all genes that are involved in the specific molecular pathways we have identified, such as Parkinson's disease and other neurodegenerative disorder pathways, to represent candidates for MR and/or associated phenotypes when hemizygous. We propose that the contribution of these candidate genes (Table 2) to many MR phenotypes can now be investigated thoroughly in mouse model systems: specifically, the 55 genes whose hemizygous deletions may be associated with MR are now amenable to study using hemizygous knockout mouse models.

Our study has exploited CNVs identified using several different platforms. As the identification technologies have improved, CNVs called using earlier technologies have been shown to over-estimate the true extent of a CNV's boundaries [47]. Thus, we expect enhanced resolution of pathogenic CNVs to also increase the power by which genic enrichments can be identified. However, it should also be noted that CNVs have been shown to affect the expression of neighbouring genes and it is possible that pathogenic CNVs may exert their genetic effect through outlying genes [48].

Finally, there is no reason why this approach can not be applied successfully to other complex neurological diseases, including schizophrenia and autism, which show a high frequency of rare *de novo* CNVs [8], [9], [49]–[51]. Many studies that are currently under-powered to demonstrate significance after correcting for multiple testing may yet prove informative of the genetic etiology of complex genomic disorders. For this, it will be crucial to collect large disease-associated CNV sets from well-phenotyped cohorts, as our analysis has shown that only then is there sufficient power to detect significant associations (Figure S2).

## Materials and Methods

### Rare *de novo* CNVs in mental retardation

For this study we collected 148 rare structural variants associated with MR from the literature, the Decipher database (https://decipher.sanger.ac.uk/), as well as from our own in-house diagnostic microarray group [52] (Table S1). The majority of these CNVs (*n* = 135, 91%) were proved to have occurred *de novo* in the patient and all were independently validated. Thirteen rare autosomal CNVs for which parental samples were unavailable were included, as were seven rare maternally inherited CNVs on the X chromosome in male patients that are considered to be as clinically relevant as *de novo* CNVs on the autosomes. Importantly, at the point of discovery none of these CNVs were known to greatly (>50%) overlap with a collection of >15,000 CNVs identified in healthy individuals as collected in the Database of Genomic Variants version 3 (http://projects.tcag.ca/variation/). All CNVs were mapped to NCBI35 coordinates. The median number of Entrez genes within a CNV was 35. Overlapping CNVs were merged to obtain a non-redundant set of 112 CNV regions (CNVRs) totalling 440 Mb of unique sequence (14.3% of the total NCBI35 human genome assembly; Table 1). CNVR sets were also formed separately from *Gain* and from *Loss* CNVs (Table 1). For 121 of the 148 CNVs, information regarding distinct anatomical or physiological abnormalities presented by the patient in addition to MR was available (Table S2). These clinical features were used to form 7 non-exclusive groupings for additional tests.

### Benign CNV datasets

We obtained 25,196 CNVs identified in 270 individuals from Redon et al. [11]. To these, we added 1,276 inherited CNVs identified in 494 individuals with a 32 k BAC tiling path array. This last set is described in Nguyen *et al.* [53] and, together with the Koolen *et al.* [52] MR–associated CNV data, are available from the Gene Expression Omnibus (http://www.ncbi.nlm.nih.gov/geo/) with accession number GSE7391. Combined, these apparently benign CNVs represent 430 Mb of unique sequence (14.0% of the total NCBI35 human genome assembly; Table 1). In the absence of information suggesting that any of the individuals present with MR, we conservatively assume that genes overlapped by these apparently benign CNVs do not contribute to the MR phenotypes.

### Genomic data sets

Assignment of protein-coding genes depended upon the particular analysis performed: for protein-coding gene counts and the Gene Ontology analysis, we assigned genes to CNVs according to Ensembl [54] (Ensembl mart version 37), whereas for KEGG pathway and MGI analyses we assigned genes to CNVs according to Entrez genes [55].

### Mouse Genome Informatics (MGI) phenotype data

Information on human NCBI genes whose mouse orthologues' disruption had been assayed were obtained from the Mouse Genome Informatics (MGI) resource (http://www.informatics.jax.org, version 3.54) [12]–[14]. We employed the MGI's human/mouse orthology and marker assignment to map MGI mouse marker phenotypes to Human Entrez genes [55]. We mapped, using unambiguous gene orthology relationships, 5,075 different MGI phenotypic annotation terms to 4,999 human genes. We considered all phenotypic annotations from all experimental methodologies described within the MGI resource. While the vast majority of these annotations are derived from the disruption of mouse genes, some phenotypes were derived from experiments in which mutant alleles are introduced into the mouse (e.g. [56]). Nonetheless, we regard the phenotypic information from these experiments as remaining informative of the biological functions or pathways to which the gene contributes. It is noted, however, that the phenotypes of all genes underlying the phenotypic enrichments we report in this work (Figure 1 and Figure 2; Table 2) were obtained through gene disruption experiments.

The MGI phenotypic annotations are categorised non-exclusively into 33 over-arching terms (Table S3). When examining finer phenotypic terms beneath an over-arching term(s) we considered only those finer terms that possessed at least 1% of the genes annotated with the over-arching term(s). This allowed a reduction in the number of tests performed thereby limiting spurious and uninformative results. The phenotypes associated with the Entrez genes overlapped by a given set of genomic regions were compared to the frequency of that phenotype across the whole genome. All *p*-values were obtained by application of the hypergeometric test and were subject to a false discovery rate (FDR) of <5% [18] (see below). Given the large number of phenotypic terms and the unrealistic assumption of terms' independence when applying an FDR, application of this significance threshold is likely to be conservative.

### Linking mouse knockout phenotypes to patient phenotypes

Many of the MR patients used in this study show additional clinical features. We tested for associations between commonly occurring non-MR clinical features in patients and a subset of MGI phenotypes. We scored patients for the presence of 7 common features derived from the London Dysmorphology Database [57]. These were: (*i*) seizures/abnormal EEG, (*ii*) facial dysmorphism, (*iii*) cleft palate, (*iv*) heart, general abnormalities, (*v*) eye abnormalities, (*vi*) brain, general abnormalities, and (*vii*) urogenital system abnormalities. Patients were excluded if specific phenotypic data were unavailable (all 19 cases from the Decipher database). As these secondary clinical feature-grouped CNVs were fewer in number than the entire set of MR–associated CNVs, and therefore relatively diminished in statistical power, the most relevant MGI phenotypic categories were selected (from a total of 33; Table S3) in order to reduce the number of tests. Two pairs of paralogous genes, *DLX1* & *DLX2* and *SELE* & *SELP*, contributed to the significant phenotypic enrichments reported within the secondary clinical feature grouped CNVs (Table 2). However, significant phenotypic enrichments that these pairs of paralogues contributed to all remained significant after removing one of the paralogous pairs (*p*<0.05; single test). Nevertheless, we note that an increased penetrance of a resulting phenotype might be expected if these pairs of paralogues provided a degree of redundancy to one another, and therefore the concurrent copy number variation of both paralogues may prove even more significant than variation involving only one [42].

### Kyoto Encyclopedia of Genes and Genomes (KEGG)

Annotations of genes involved in neurodegenerative pathways were obtained from KEGG [17]. KEGG genes were collated if they belonged to KEGG Pathways section 5.3, namely Alzheimer's disease (KEGG pathway 05010), Parkinson's disease (KEGG pathway 05020), Amyotrophic Lateral Sclerosis (KEGG pathway 05030), Huntington's disease (KEGG pathway 05040), Dentatorubropallidoluysian atrophy (KEGG pathway 05050) and Prion Diseases (KEGG pathway 05060). KEGG genes were mapped to NCBI Entrez genes using associations provided by KEGG.

### Tissue expression of genes

For human gene expression data, we used GNF's gene atlas data for the MAS5-condensed human U133A and GNF1H chips, considering all 74 non-cancer tissues [16]. Expression levels were mapped to LocusLink identifiers and to 11,594 Ensembl Ensmart 37 (NCBI35) genes using the annotation tables supplied by GNF. To identify genes that are highly expressed in the brain we selected those genes whose expression in the whole brain exceeded by 4-fold their median expression in all other non-brain tissues after excluding cancerous tissues. This resulted in 435 genes (3.75%) being classified as exhibiting strong expression in the brain relative to other tissues. However, the significant enrichments reported in the Results were also found when brain-specificity was redefined at 2-, 3-, 7-, 10-, 11-, 12-, 13-, and 14-fold expression in the brain above the median across all other tissues.

### Postsynaptic protein genes

A set of postsynaptic protein genes was obtained from Collins *et al.* [58] and matched to human orthologues using Ensembl Compara [59]. Over- or under-representation of these genes within human CNVs was assessed using the hypergeometric distribution and all human Ensembl genes as the background set.

### Statistical tests

The significance of enrichments or deficits of genes associated with particular MGI knockout phenotypes, genes involved in KEGG neurodegenerative pathways, genes associated with particular GO terms and brain-specific genes were evaluated using hypergeometric tests. Where multiple tests were performed, a False Discovery Rate (FDR) multiple testing correction was applied to ensure a less than 5% likelihood of any significant term being a false-positive [18]. Explicitly, an FDR correction was applied when testing for enrichments of genes: (*i*) associated with MGI phenotypic terms, (*ii*) belonging to individual KEGG neurodegenerative pathways or (*iii*) annotated with Gene Ontology terms (Figure S1). All other tests performed were single tests.

Calculation of the fold-enrichment within MR–associated CNVs for the final set of 55 MR–associated candidate genes was performed by random sampling. 1000 gene sets, matched in gene number to that within the *Loss* MR–associated CNVRs, were obtained by random sampling and the median expected number of genes, 23 (*std.dev.* = 4.6), annotated with one or more significantly-enriched terms (Figure 1 and Figure 2) was recorded. Given the 50 candidate genes within the *Loss* CNVRs, we thus estimate a ∼2.2-fold enrichment over the number expected by chance.

## Supporting Information

Figure S1 Gene Ontology *Slim* terms significantly enriched among genes within MR–associated CNVRs. MR–associated CNVR sets denoted “minus benign CNVs” have had genes removed that are also overlapped by benign CNVRs showing the same direction of copy number change (i.e. *Gain* or *Loss*) as its overlapping MR–associated CNVR. We tested whether genes within MR–associated CNVRs exhibit a bias towards specific molecular and cellular functions using a reduced set of Gene Ontology (GO) annotations, namely *GOslim* terms [15],[60]. Columns marked with an asterisk (“*”) are associated with significant differences over expected values after application of an FDR of 5%. The Gene Ontology Consortium's [15],[60], annotations mapped to Ensembl genes were obtained from the Ensembl Ensmart 37 database [54],[59]. To reduce the number of terms examined and the loss of significance arising from multiple-testing, only *GOSlim* terms (a subset of GO terms: 53 process, 41 function and 36 component terms) were considered. Of 9 significantly over-represented GOSlim terms, 7 were related to DNA-binding, DNA metabolism or transcription regulation, with nuclear localisation being the only cellular component significantly enriched (*p* = 3.4×10^−5^). The remaining 2 over-represented terms, *Intracellular* and *Binding*, could also be attributed to this DNA-associated signal. Despite its small size, the *Gain* MR–associated CNVR data set was significantly enriched in genes with nucleic acid binding functions (+23%, *p* = 5×10^−4^) and transcription (+26%, *p* = 2×10^−3^), as indeed was the *Loss* data set. By contrast, benign CNV genes show significant tendencies to encode proteins with roles in immunity and host defense [20],[21].
(0.04 MB PDF)

Figure S2 A high percentage of the entire MR–associated CNV set is required for the reported enrichments to reach significance; this demonstrates the collective power of a community's data set. Shown is the percentage of CNVs required from the total number of CNVs collated for this study (*n* = 148) to reach significance for five annotations: namely, the mouse orthologue's knock-out phenotypes of (*i*) “nervous system”, (*ii*) “abnormal axon morphology” and (*iii*) “abnormal dopaminergic neuron morphology”, together with (*iv*) KEGG Neurogenerative disease and (*v*) Parkinson's disease pathway genes. For each of 13 different proportions of the entire CNV dataset, we randomly sampled 100 sets of MR–associated CNVs. We then recorded the number of sets at that particular coverage that yielded a significant enrichment for each of the 5 annotations for *Loss* CNVs. Crucially, the significant enrichment of the “nervous system” phenotype genes was obtained only, on average, with 99% (147/148) of the CNVs. The two finer-scale MGI phenotypes, “abnormal axon morphology” and “abnormal dopaminergic neuron morphology” were obtained, on average, with ∼65% and ∼85% of the CNVs, respectively, while the two KEGG disease pathway enrichments gain significance at 45%–55% coverage. These results illustrate the data set sizes required to confidently detect these signals and hence the value of collating disparate data sets.
(0.05 MB PDF)

Table S1 Sources of MR–associated CNVs employed in this study. For each of the 17 sources of CNVs, the publication, number of CNVs obtained, experimental platform used to discover the CNVs, along with the platform's approximate resolution, and the broadness of the phenotype of the patients studied, are provided.
(0.09 MB PDF)

Table S2 MR patient phenotypes and their individual CNVs. All CNVs used in this study are listed together with the clinical features of the relevant patient. CNVs from Decipher are not listed with clinical information as they do not refer to a specific individual but to a collection. All CNVs are confirmed *de novo* unless indicated with an asterisk (*). Note that the CNV numbering is not sequential as 6 CNVs from Koolen *et al.* (Table S1) were found later after further quality control checks to be inherited and thus were removed from consideration. For extended reference details, please see Table S1.
(0.20 MB PDF)

Table S3 Matching patients' secondary clinical features to MGI mouse phenotype categories. For each set of CNVs grouped by secondary clinical features, the MGI phenotypic categories tested against are shown with an ‘X’. As CNVs grouped by secondary clinical features are subsets of the entire set of MR–associated CNVs, we sought to limit the number of statistical tests performed by considering only a subset of all MGI phenotypic terms. Thus, one of us (BVD) selected the most relevant categories (from a total of 33) of MGI phenotypic terms that only then were tested for significant enrichments.
(0.13 MB PDF)
