# Supplementary figures and images for: Forging Links between Human Mental Retardation–Associated CNVs and Mouse Gene Knockout Models
Source: PLoS Genet. 2009 Jun 26;5(6):e1000531. doi: 10.1371/journal.pgen.1000531 (PMC2694283; doi:10.1371/journal.pgen.1000531)

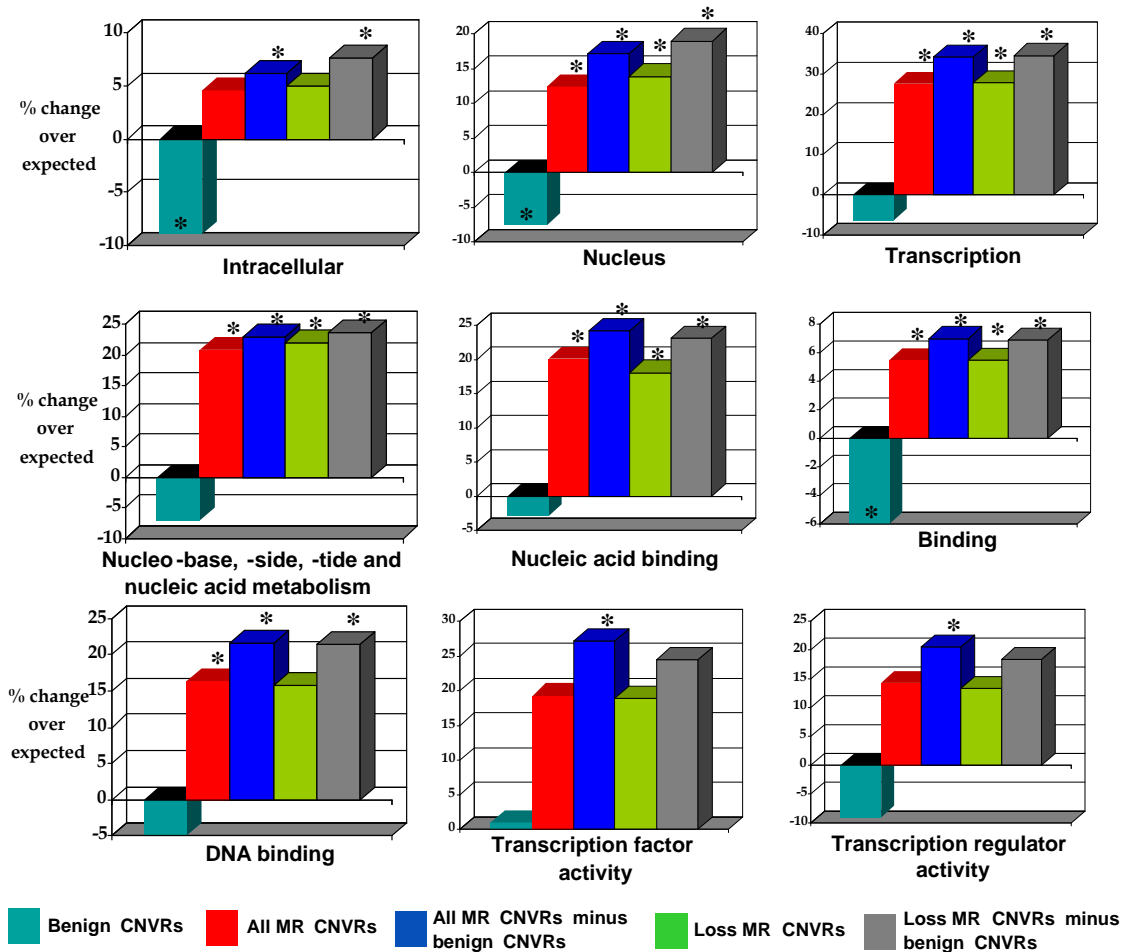

Supplement: Figure S1 — Gene Ontology Slim terms significantly enriched among genes within MR–associated CNVRs. MR–associated CNVR sets denoted “minus benign CNVs” have had genes removed that are also overlapped by benign CNVRs showing the same direction of copy number change (i.e. Gain or Loss) as its overlapping MR–associated CNVR. We tested whether genes within MR–associated CNVRs exhibit a bias towards specific molecular and cellular functions using a reduced set of Gene Ontology (GO) annotations, namely GOslim terms [15],[60]. Columns marked with an asterisk (“*”) are associated with significant differences over expected values after application of an FDR of 5%. The Gene Ontology Consortium's [15],[60], annotations mapped to Ensembl genes were obtained from the Ensembl Ensmart 37 database [54],[59]. To reduce the number of terms examined and the loss of significance arising from multiple-testing, only GOSlim terms (a subset of GO terms: 53 process, 41 function and 36 component terms) were considered. Of 9 significantly over-represented GOSlim terms, 7 were related to DNA-binding, DNA metabolism or transcription regulation, with nuclear localisation being the only cellular component significantly enriched (p = 3.4×10^−5^). The remaining 2 over-represented terms, Intracellular and Binding, could also be attributed to this DNA-associated signal. Despite its small size, the Gain MR–associated CNVR data set was significantly enriched in genes with nucleic acid binding functions (+23%, p = 5×10^−4^) and transcription (+26%, p = 2×10^−3^), as indeed was the Loss data set. By contrast, benign CNV genes show significant tendencies to encode proteins with roles in immunity and host defense [20],[21]. (0.04 MB PDF) [file pgen.1000531.s001.pdf]

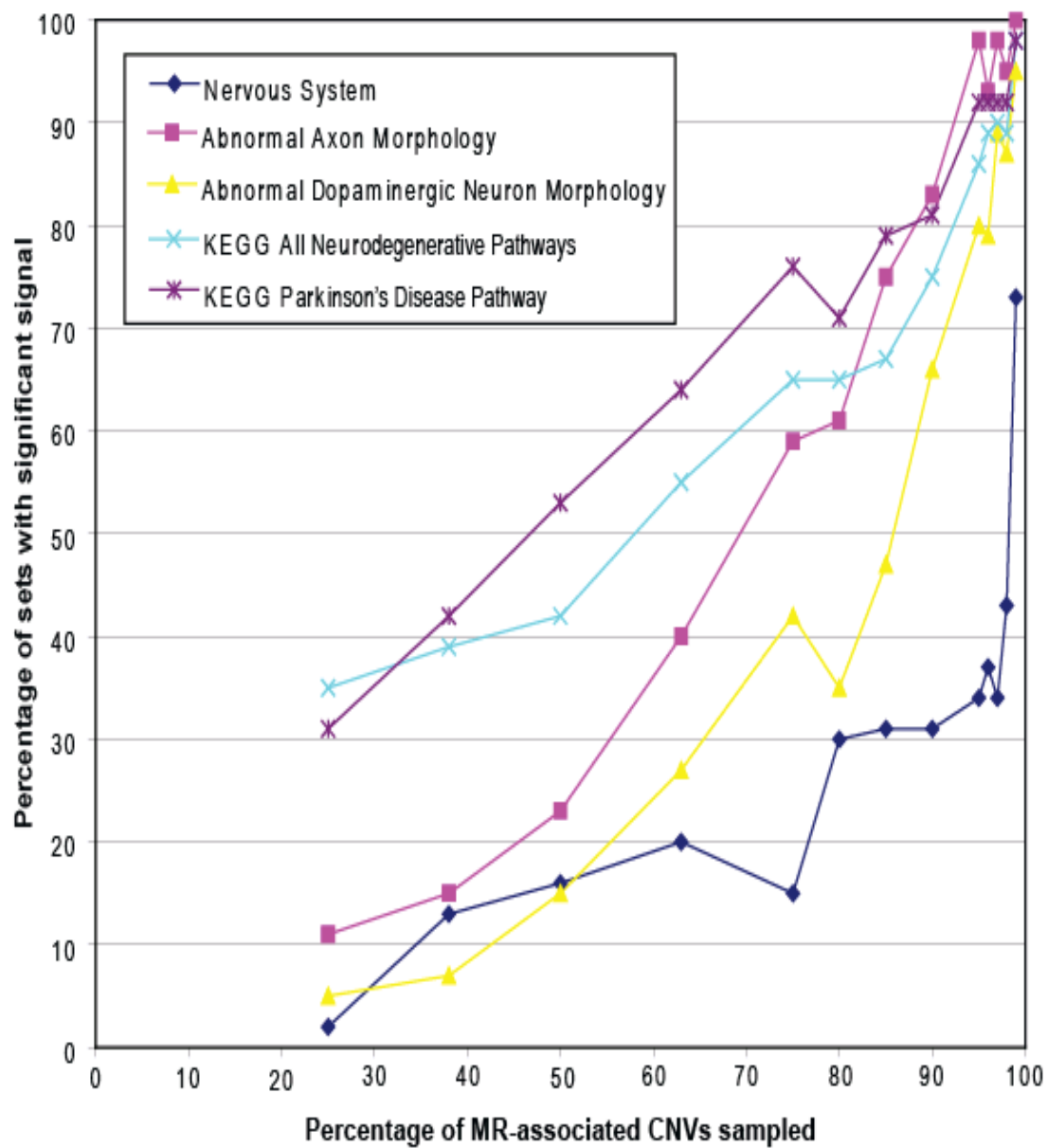

Supplement: Figure S2 — A high percentage of the entire MR–associated CNV set is required for the reported enrichments to reach significance; this demonstrates the collective power of a community's data set. Shown is the percentage of CNVs required from the total number of CNVs collated for this study (n = 148) to reach significance for five annotations: namely, the mouse orthologue's knock-out phenotypes of (i) “nervous system”, (ii) “abnormal axon morphology” and (iii) “abnormal dopaminergic neuron morphology”, together with (iv) KEGG Neurogenerative disease and (v) Parkinson's disease pathway genes. For each of 13 different proportions of the entire CNV dataset, we randomly sampled 100 sets of MR–associated CNVs. We then recorded the number of sets at that particular coverage that yielded a significant enrichment for each of the 5 annotations for Loss CNVs. Crucially, the significant enrichment of the “nervous system” phenotype genes was obtained only, on average, with 99% (147/148) of the CNVs. The two finer-scale MGI phenotypes, “abnormal axon morphology” and “abnormal dopaminergic neuron morphology” were obtained, on average, with ∼65% and ∼85% of the CNVs, respectively, while the two KEGG disease pathway enrichments gain significance at 45%–55% coverage. These results illustrate the data set sizes required to confidently detect these signals and hence the value of collating disparate data sets. (0.05 MB PDF) [file pgen.1000531.s002.pdf]
