# Supplementary material for: Forging Links between Human Mental Retardation–Associated CNVs and Mouse Gene Knockout Models
Source: PLoS Genet. 2009 Jun 26;5(6):e1000531. doi: 10.1371/journal.pgen.1000531 (PMC2694283; doi:10.1371/journal.pgen.1000531)
Supplement: Table S1 — Sources of MR–associated CNVs employed in this study. For each of the 17 sources of CNVs, the publication, number of CNVs obtained, experimental platform used to discover the CNVs, along with the platform's approximate resolution, and the broadness of the phenotype of the patients studied, are provided. (0.09 MB PDF) [file pgen.1000531.s003.pdf]

Study	Reference	Number of patients	Number of MR CNVs	Group	Targets	Number of targets	Mean spacing of targets	Follow-up studies
1	Vissers *et al*. (*1*)	20	2	MR and additional dysmorphisms (Checklist score >3)*	BAC	3,569	1 Mb	FISH
2	Shaw-Smith *et al*. (*2*)	50	7	Learning disability and dysmorphisms	BAC	3,500	1 Mb	FISH, genotyping
3	de Vries *et al*. (*3*)	100	11	MR with/or without congenital malformations and/or dysmorphism	BAC	32,447	100 kb	FISH, MLPA
4	Schoumans *et al*. (*4*)	41	4	MR (mild to severe) and dysmorphic features, malformations, and/or a family history (Checklist score >3)*	BAC	2,600	1 Mb	FISH
5	Tyson *et al*. (*5*)	22	2	MR (mild to severe) and nonsyndromic pattern of dysmorphic features	BAC	1,003 or 2,600	1 Mb, 3 Mb	FISH, microsatellite DNA analysis, real-time PCR
6	Menten *et al*. (*6*)	140	12	MR with one or more major congenital malformation or dysmorphism, or both	BAC	3,431	1 Mb	FISH, RT qPCR
7	Miyake *et al*. (*7*)	30	2	MR associated with some dysmorpic features	BAC	2,173	1.5 Mb	FISH
8	Friedman *et al*. (*8*)	100	10	MR (moderate-severe) and > additional clinical features: one major malformation, microcephaly, abnormal growth, or multiple minor anomalies	oligo	100,000	30 kb	FISH
9	Krepischi-Santos *et al*. (*9*)	95	13	Syndromic phenotype; almost all patients presented MR	BAC	3500	1 Mb	FISH, MLPA
10	Rosenberg *et al*. (*10*)	81	7	MR (mild to severe) and cranial/facial dysmorphisms and at least one additional dysmorphic feature	BAC	3500	1 Mb	FISH, MAPH
11	Engels *et al*. (*11*)	60	3	MR mostly combined with congenital anomalies	BAC	6000 or 8000	500 kb	FISH
12	Hoyer *et al*. (*12*)	104	10	MR, with or without multiple congenital anomalies	oligo	100,000	30 kb	FISH, qPCR for selected deletions <100kb and dup
13	Fan *et al*. (*13*)	100	6	MR (Checklist >3)*	oligo	44,290	30-35 kb	FISH, BaCGH or OaCGH244K;
14	Aradhya *et al*. (*14*)	20	10	MR and dysmorphic features or congenital anomalies or growth retardation	oligo, BAC	44,290, 2,600	30-35 kb, 1 Mb	FISH
15	Svensson *et al*. (*15*)		1	MR and skeletal abnormalities	oligo	44,290, 2,600	30-35 kb, 1 Mb	FISH
16	Decipher (*16*)		19	Syndromic phenotype including MR				
17	Koolen *et al*. (*17*)	386	29	MR with/or without congenital malformations and/or dysmorphism	BAC	32,447	100 kb	FISH, MLPA
	**Total**	**1349**	148					

(* checklist scoring based on de Vries *et al*, (*18*))

References:

1. Vissers LE, van Ravenswaaij CM, Admiraal R, Hurst JA, de Vries BB, et al. (2004) Mutations in a new member of the chromodomain gene family cause CHARGE syndrome. Nat Genet 36: 955-957.
2. Shaw-Smith C, Redon R, Rickman L, Rio M, Willatt L, et al. (2004) Microarray based comparative genomic hybridisation (array-CGH) detects submicroscopic chromosomal deletions and duplications in patients with learning disability/mental retardation and dysmorphic features. J Med Genet 41: 241-248.
3. de Vries BB, Pfundt R, Leisink M, Koolen DA, Vissers LE, et al. (2005) Diagnostic genome profiling in mental retardation. Am J Hum Genet 77: 606-616.
4. Schoumans J, Ruivenkamp C, Holmberg E, Kyllerman M, Anderlid BM, et al. (2005) Detection of chromosomal imbalances in children with idiopathic mental retardation by array based comparative genomic hybridisation (array-CGH). J Med Genet 42: 699-705.
5. Tyson C, Harvard C, Locker R, Friedman JM, Langlois S, et al. (2005) Submicroscopic deletions and duplications in individuals with intellectual disability detected by array-CGH. Am J Med Genet A 139: 173-185.
6. Menten B, Maas N, Thienpont B, Buysse K, Vandesompele J, et al. (2006) Emerging patterns of cryptic chromosomal imbalance in patients with idiopathic mental retardation and multiple congenital anomalies: a new series of 140 patients and review of published reports. J Med Genet 43: 625-633.
7. Miyake N, Matsumoto N (2005) [Microarray CGH]. Nippon Rinsho 63 Suppl 12: 167-170.
8. Friedman JM, Baross A, Delaney AD, Ally A, Arbour L, et al. (2006) Oligonucleotide microarray analysis of genomic imbalance in children with mental retardation. Am J Hum Genet 79: 500-513.
9. Krepischi-Santos AC, Vianna-Morgante AM, Jehee FS, Passos-Bueno MR, Knijnenburg J, et al. (2006) Whole-genome array-CGH screening in undiagnosed syndromic patients: old syndromes revisited and new alterations. Cytogenet Genome Res 115: 254-261.
10. Rosenberg C, Knijnenburg J, Bakker E, Vianna-Morgante AM, Sloos W, et al. (2006) Array-CGH detection of micro rearrangements in mentally retarded individuals: clinical significance of imbalances present both in affected children and normal parents. J Med Genet 43: 180-186.
11. Engels H, Brockschmidt A, Hoischen A, Landwehr C, Bosse K, et al. (2007) DNA microarray analysis identifies candidate regions and genes in unexplained mental retardation. Neurology 68: 743-750.
12. Hoyer J, Dreweke A, Becker C, Gohring I, Thiel CT, et al. (2007) Molecular karyotyping in patients with mental retardation using 100K single-nucleotide polymorphism arrays. J Med Genet 44: 629-636.
13. Fan YS, Jayakar P, Zhu H, Barbouth D, Sacharow S, et al. (2007) Detection of pathogenic gene copy number variations in patients with mental retardation by genomewide oligonucleotide array comparative genomic hybridization. Hum Mutat 28: 1124-1132.
14. Aradhya S, Manning MA, Splendore A, Cherry AM (2007) Whole-genome array-CGH identifies novel contiguous gene deletions and duplications associated with

developmental delay, mental retardation, and dysmorphic features. Am J Med Genet A 143: 1431-1441.

15. Svensson AM, Curry CJ, South ST, Whitby H, Maxwell TM, et al. (2007) Detection of a de novo interstitial 2q microdeletion by CGH microarray analysis in a patient with limb malformations, microcephaly and mental retardation. Am J Med Genet A 143A: 1348-1353.
16. DECIPHER DatabasE of Chromosomal Imbalance and Phenotype in Humans using Ensembl Resources. https://decipher.sanger.ac.uk/.
17. Koolen DA, Pfundt R, de Leeuw N, Hehir-Kwa JY, Nillesen WM, et al. (2009) Genomic microarrays in mental retardation: a practical workflow for diagnostic applications. Hum Mutat 30: 283-292.
18. de Vries BB, White SM, Knight SJ, Regan R, Homfray T, et al. (2001) Clinical studies on submicroscopic subtelomeric rearrangements: a checklist. J Med Genet 38: 145-150.
