# Supplementary material for: Forging Links between Human Mental Retardation–Associated CNVs and Mouse Gene Knockout Models
Source: PLoS Genet. 2009 Jun 26;5(6):e1000531. doi: 10.1371/journal.pgen.1000531 (PMC2694283; doi:10.1371/journal.pgen.1000531)
Supplement: Table S2 — MR patient phenotypes and their individual CNVs. All CNVs used in this study are listed together with the clinical features of the relevant patient. CNVs from Decipher are not listed with clinical information as they do not refer to a specific individual but to a collection. All CNVs are confirmed de novo unless indicated with an asterisk (*). Note that the CNV numbering is not sequential as 6 CNVs from Koolen et al. (Table S1) were found later after further quality control checks to be inherited and thus were removed from consideration. For extended reference details, please see Table S1. (0.20 MB PDF) [file pgen.1000531.s004.pdf]

Case	Loss / Gain	Chr	Location	Start position	End position	Genomic size	Reference	Clinical features
1	loss	1	1pter-1p36.31	0	6,466,179	6,466,179	Rosenberg et al. 2006	MR, epilepsy, FD, shortening of metacarpals and metatarsals, hirsutism
2	loss	1	1p36pter	18,367	5,259,637	5,241,270	Decipher database	No clinical data available
3	loss	1	1p36.33	536,213	1,667,191	1,130,978	Shaw Smith et al. 2004	MR, microcephaly, short stature, FD, seizures
4	loss	1	1p36.31p36.32	2,818,104	6,466,179	3,648,076	Menten et al. 2006	MR, microcephaly, ventricular septum defect, large corneae, midface hypoplasia, presacral groove
5	loss	1	1p36.32	2,818,104	3,855,092	1,036,989	Krepischi-Santos 2006	MR, seizures, FD, coloboma right iris and choroid, 5th finger clinodactyly, hypoplasia labia minora and anteriorly displaced anus, patent ductus arteriosus and dextrocardia.
6	loss	1	1p36.21p36.31	6,579,079	14,834,824	8,255,745	Aradhya, 2007	MR, growth deficiency, microcephaly
7	gain	1	1p36.11-p12	23,879,343	27,925,539	4,046,196	Shaw Smith et al. 2004	MR, microcephaly
8	loss	1	1p36.11p35.3	27,103,889	28,002,995	899,106	Koolen et al. 2008	MR, FD, congenital cataract, atrofic cerebri, abnormal shape of the skull
9	loss	1	1p34.3p34.2	39,219,410	43,071,596	3,852,186	de Vries et al. 2005	MR, intrauterine growth retardation, general dystrophy, microcephaly, FD, seizures, hypomyelinisation cerebrum
10	loss	1	1p31.1-p31.3	61,713,855	75,751,197	14,037,342	Shaw Smith et al. 2004	MR, microcephaly, cleft palate
11*	gain	1	1q21.1q21.1	143,533,957	145,377,353	1,843,396	de Vries et al. 2005	MR, FD, growth retardation
12	loss	1	1q24.1q24.2	162,334,119	166,583,262	4,249,144	Engels et al. 2007	severe MR, seizures, reduced sense of pain, FD, small dysplastic ears, 5th finger clinodactyly
13	loss	1	1q41q42.12	217,728,706	220,721,168	2,992,463	Krepischi-Santos 2006	MR, short stature, hypotonia, hemiparesia at right, cleft palate, micropenis, hemivertebrae.
14*	loss	1	1q43q44	238,361,436	242,233,562	3,872,126	Koolen et al. 2008	moderate MR, hypotonia, epilepsy, FD, growth retardation (pre- and postnatal), microcephaly, phimosis, scoliosis, hypoplasia corpus callosum and wide liquor spaces
15	loss	1	1q44q44	239,927,998	243,390,088	3,462,090	Koolen et al. 2008	severe MR, epilepsy, hypotonia, FD,

								microcephaly, delayed myelinisation and hypoplasia, corpus callosum, hirsutism
16	loss	1	1q44q44	240,163,587	241,960,518	1,796,931	Koolen et al. 2008	severe MR, epilepsy, FD, growth retardation (pre- and postnatal), microcephaly, coloboma of retina/choroid, clinodactyly 5th fingers
17	loss	2	2p16.3p16.3	48,083,811	49,588,514	1,504,703	Koolen et al. 2008	severe MR, hypotonia, epilepsy, FD, growth retardation (postnatal), microcephaly, microphthalmia
18	loss	2	2p16.3	48,573,973	52,841,386	4,267,413	Krepischi-Santos 2006	MR, joint hyperextensibility with tendency to dislocation suggestive of connective tissue disease.
19	loss	2	2p16.3	50,857,428	51,178,791	321,363	Friedman et al. 2006	mild MR, Asperger syndrome, FD, scoliosis with 13 ribs on left, bifid right second rib, hemivertebrae and fusions
20	loss	2	2p16.1p15	57,874,237	61,472,359	3,598,122	Koolen et al. 2008	MR, epilepsy, growth retardation, microcephaly, hypotonia, FD, clinodactyly fifth finger, hirsutism
21*	gain	2	2p13p14	66,555,163	70,714,771	4,159,608	Koolen et al. 2008	MR, growth retardation (prenatal), pectus excavatum
22	loss	2	2q14.1q14.3	115,776,113	129,545,173	13,769,060	Fan, 2007	MR, seizures, FD, hypoplastic teeth, pectus excavatum, extra nipples, facial telangiectasias, brachydactyly
23	loss	2	2q14.3q22.1	123,919,808	141,066,577	17,146,769	Fan, 2007	severe MR, low birth weight, nephrolithiasis as newborn, FD, microgenitalism, contractures of fingers, fusion 2-3rd toes, nail hypoplasia, clinical diagnosis of arthrogryposis type 5.
24	loss	2	2q22.3q24.1	144,857,811	159,023,030	14,165,219	Schoumans et al. 2006	MR, FD, postnatal growth retardation, seizures, microcephaly, anal atresia, heart malformation
25	loss	2	2q22.3-2q23.2	145,542,903	149,909,044	4,366,141	Vissers et al. 2003	severe MR, FD, microcephaly, short stature, epilepsy, short fifth fingers
26	loss	2	2q23.1q23.2	149,174,652	150,093,789	919,137	de Vries et al. 2005	severe MR, FD, epilepsy, short fifth fingers
27	loss	2	2q24.3q31.1	166,623,782	176,436,770	9,812,988	Schoumans et al. 2006	MR, FD, seizures, cleft palate, prenatal growth retardation, syndactyly, camptodactyly
28	loss	2	2q31.1q31.1	174,393,697	176,403,966	2,010,269	Svensson et al., 2007	MR, microcephaly

30	loss	2	2q32.2	196,763,193	204,932,469	8,169,276	Decipher database	No clinical data available
31	loss	2	2q37	239,791,932	242,742,892	2,950,960	Decipher database	No clinical data available
32	loss	3	3p24.3-p24.1	20,232,288	30,442,871	10,210,583	Rosenberg et al. 2006	mild MR, FD, club feet, triphalangeal thumbs, mild anemia
33	loss	3	3p21p21	50,129,819	51,974,846	1,845,028	Miyake 2006	MR, growth retardation, hypotonia, FD, left Sprengel deformity, short fingers, simian crease, micropenis
34*	loss	3	3p14.2p14.1	63,643,643	65,070,262	1,426,619	Koolen et al. 2008	severe MR, FD, growth retardation, cryptorchidism
37*	loss	3	3q26.1q26.1	165,392,008	165,644,219	252,211	Koolen et al. 2008	MR, FD, microcephaly, small scrotum, arachnodatyly, nephrotic syndrome, joint contractures,
38	loss	3	3q27.1q29	184,307,209	196,730,725	12,423,516	de Vries et al. 2005	MR, low birth weight, short length (-4SD), FD, hypogenitalism, bifid scrotum, double row of teeth, hypoplastic kidneys and mixed conductive/perceptive deafness.
39	loss	3	3q29	197,239,138	198,903,881	1,664,744	Decipher database	No clinical data available
40	loss	3	3q29	197,843,365	199,032,077	1,188,712	Krepischi-Santos 2006	moderate MR, growth retardation (pre- and postnatal), microcephaly, FD, myopia, simian palmar crease left
41	loss	4	4pter	59,384	1,944,179	1,884,795	Decipher database	No clinical data available
42	gain	4	4p16.13q16.13	848,236	2,088,582	1,240,346	Koolen et al. 2008	MR, FD, iris coloboma, hypotonia, perceptive/conductive hearing loss, anal atresia
43*	gain	4	4p16.3p16.3	947,079	1,610,777	663,698	Koolen et al. 2008	mild MR, hypotonia, FD, microcephaly,
44	loss	4	4p16.2p16.3	1,444,179	8,683,198	7,239,020	Schoumans et al. 2006	MR, FD, growth retardation (pre- and postnatal), microcephaly, seizures
45	loss	4	4p12p13	44,846,185	46,301,417	1,455,232	Engels et al. 2007	Severe MR, seizures, FD, limb spasticity, hypotonia, brain atrophy with ventriculomegaly, hypoplastic corpus callosum, kyphoscoliosis, inverted nipples, hirsutism
46	loss	4	4q21.21-q22.1	82,146,749	93,214,433	11,067,684	Friedman et al. 2006	severe MR, extreme short stature, severe hypotonia, FD, small hands and feet, scoliosis
47	loss	4	4q24q24	103,206,374	106,421,015	3,214,641	Koolen et al. 2008	MR, FD, postaxial polydactyly, supernumerary teeth

48	loss	4	4q31.21q31.23	146,280,874	151,907,509	5,626,635	Aradhya, 2007	MR, speech delay, cleft lip and palate, heart defect, vertebral anomalies
49	loss	5	5pter	82,978	11,647,390	11,564,412	Decipher database	No clinical data available
50	loss	5	5q14.3-15	87,975,410	93,668,872	5,693,462	Hoyer, 2007	MR, corpus callosum hypoplasia
51	loss	5	5q35.1q35.1	170,496,421	171,659,856	1,163,435	Menten et al. 2006	MR, FD, tetralogy of Fallot, double outlet right ventricle
52	gain	5	5q35.1q35.1	170,524,984	171,521,158	996,174	de Vries et al. 2005	MR, semilobular holoprosencephaly, finger-like thumbs, polydactyly
53	loss	5	5q35	175,110,935	177,297,123	2,186,188	Decipher database	No clinical data available
54	loss	5	5q35	175,372,550	177,298,016	1,925,466	Krepischi-Santos 2006	MR, FD, absent left kidney
55	gain	6	6p12.3	49,186,791	50,305,314	1,118,523	Rosenberg et al. 2006	MR, FD, hypotonia, joint hyperlaxity
56	loss	6	6q11.1q13	62,019,167	73,157,911	11,138,744	Engels et al. 2007	MR, FD, slight brain anomaly, pes valgus, frequent infections
57*	gain	6	6q15q15	90,930,579	91,417,427	486,848	Koolen et al. 2008	MR, FD, short hands with broad fingers, multicystic kidney
58	loss	6	6q16.1-21	99,645,914	112,529,340	12,883,426	Hoyer, 2007	MR, double outlet right ventricle
59*	gain	6	6q24.1q24.1	141,140,329	142,710,602	1,570,273	Koolen et al. 2008	Severe MR, autism, macrocephaly
60	loss	7	7p22.3-22.2	2,678,041	4,257,670	1,579,629	Hoyer, 2007	MR, cerebral palsy, scoliosis, Tetralogy of Fallot
61	loss	7	7p22.1-p22.2	3,304,850	6,940,933	3,636,083	Friedman et al. 2006	severe MR, FD, growth retardation (pre- and postnatal), microcephaly, patent ductus arteriosus, ventricular septal defect, 2-3 syndactyly of toes
62*	gain	7	7p13p13	43,785,108	43,955,482	170,374	Koolen et al. 2008	severe MR, microcephaly, hydronephrosis
63	loss	7	7q11.21-q11.23	65560878	74,365,025	8,804,147	Vissers et al. 2003	Severe MR, Microcephaly, FD, pulmonary arterial and valve stenosis
64	loss	7	7q11.23	71870262	73,965,226	2,094,964	Decipher database	No clinical data available
66*	gain	7	7q21.13q21.13	87987971	89,444,918	1,456,947	Koolen et al. 2008	mild MR, FD
67	loss	7	7q22.3q22.1	96827950	100,269,227	3,441,277	Koolen et al. 2008	Moderate MR
68	loss	7	7q22.1q22.3	102,327,975	104,331,051	2,003,076	Krepischi-Santos 2006	mild MR, abdominal obesity, hyperphagia, hypothyroidism
69	gain	7	7q36.3	155,668,694	159,882,622	4,213,928	Tyson et al. 2006	MR, FD, microcephaly, long thin fingers, rotated thumbs, 2-3 toe syndactyly, hirsutism, small optic disc, retinal pigmentation, myopia
70	loss	8	8p23.1	8,242,028	11,715,250	3,473,222	Decipher database	No clinical data available
71	loss	8	8q22.1q2	93,893,266	97,183,101	3,289,835	Aradhya,	MR, FD, camptodactyly

			2.1				2007	
73	loss	9	9q22.3	92,905,637	99,842,585	6,936,949	Shaw Smith et al. 2004	severe MR, overgrowth, craniosynostosis, macrocephaly
74	loss	9	9q31.1q31.1	99,737,286	102,584,525	2,847,239	de Vries et al. 2005	moderate MR, FD, microcephaly, transposition of the great vessels
75	loss	9	9q33.1q33.1	115,301,965	115,783,506	481,541	de Vries et al. 2005	mild MR, FD, macrocephaly, autism
76	loss	9	9q34-q34	137,668,343	138,774,031	1,105,688	Menten et al. 2006	MR, hypotonia, abdominal muscle hypoplasia, fine hair, macroglossia
77	loss	10	10q22.3q23.2	81,860,757	88,860,757	7,000,000	Koolen et al. 2008	mild MR, FD
78	loss	10	10q25-q26.11	111,018,364	120,061,007	9,042,643	Menten et al. 2006	Autism, macrocephaly, strabismus, oral frenulae, carpal synostosis
79	loss	11	11p11.2	44,000,709	46,025,315	2,024,606	Decipher database	No clinical data available
80	gain	11	11q12.3-q13.1	61,859,463	65,310,025	3,450,563	Tyson et al. 2006	moderate MR, FD, growth retardation (pre- and postnatal), microcephaly, campto/brachy/clinodactyly fifth fingers, kyphoscoliosis
81	loss	11	11q14.1q14.1	77,796,262	85,078,012	7,281,750	de Vries et al. 2005	mild MR, FD
82	loss	12	12q14.2-q15	63,342,649	66,780,095	3,437,446	Friedman et al. 2006	mild MR, growth retardation, partial anodontia, mild limitation of extension at elbows, mildly short and narrow fingers, tremor, osteopoikilosis
83	gain	12	12q24.21q24.23	114,909,852	116,868,012	1,958,160	de Vries et al. 2005	Severe MR, FD, growth retardation (pre- and postnatal), microcephaly, behavioral problems
84	loss	13	13q12.11-q12.13	18,867,056	24,517,730	5,650,674	Friedman et al. 2006	moderate MR, growth retardation, microcephaly, café au lait spots, anemia, thrombocytopenia, neutropenia.
85	gain	13	13q31.3-q33.1	88,942,610	101,953,069	13,010,459	Menten et al. 2006	MR, trigonocephaly, strabismus, nasal speech, deep hoarse voice, broad thumbs
86	loss	13	13q32.3	99,897,068	101,953,069	2,056,001	Rosenberg et al. 2006	MR, FD, tall stature, corpus callosum agenesis, hearing loss
87	loss	14	14q11.2	19,584,863	21,207,935	1,623,072	Friedman et al. 2006	MR, overgrowth (pre- and postnatal), hypotonia, FD, hypermobility, long toes with 2-3 syndactyly
88	loss	14	14q11.2	20,741,117	20,918,741	177,624	Friedman et al. 2006	MR, FD, ventricular septal defect, large patent ductus arteriosus, plagiocephaly, undescended testes and hypoplastic scrotum
89	loss	15	15q11.2q11.2	20,335,887	21,208,435	872,548	Aradhya, 2007	MR, lissencephaly, ptosis, eczema

90	loss	15	15q11.2q13.1	20,335,887	26,199,055	5,863,168	Aradhya, 2007	MR, minor anomalies, posthemorrhagic hydrocephalus
91	loss	15	15q11q13	20,473,731	24,422,558	3,948,827	Decipher database	No clinical data available
92*	gain	15	15q11.2q13.1	21,162,020	26,584,823	5,422,803	Koolen et al. 2008	MR, epilepsy, autism, FD, growth retardation, microcephaly
93	loss	15	15q24.1q24.2	70,764,625	73,807,021	3,042,396	Fan, 2007	severe MR, abnormal dentition, right ventricular arrhythmia, thin hair, extra row teeth, hypotonia, abnormal nose
94*	loss	15	15q24.1q24.2	72,197,367	73,735,634	1,538,267	de Vries et al. 2005	MR, FD, hypospadia, microcephaly, growth retardation (pre- and postnatal), small hands
95	gain	16	16p13.3	925,718	3,864,938	2,939,220	Friedman et al. 2006	moderate MR, FD, pectus excavatum, C5-C6 vertebral fusion, pes cavus and clawed toes
96	gain	16	16p13.2-p13.3	2,882,187	8,526,890	5,644,703	Menten et al. 2006	MR, FD, median cleft palate, small penis
97	loss	16	16p13	3,742,473	3,781,450	38,978	Decipher database	No clinical data available
98	loss	16	16p12.1	24,061,164	24,919,925	858,761	Hoyer, 2007	MR
99	gain	17	17pter-p13.3	0	1,200,000	1,200,000	Rosenberg et al. 2007	mild MR, FD
100	loss	17	17p13.3	58,380	2,402,100	2,343,720	Decipher database	No clinical data available
101	loss	17	17p11.2p11.2	14,916,513	16,800,973	1,884,461	Schoumans et al. 2006	MR, FD, growth retardation (post natal), overweight, behavioural problems, sleeping disturbance
102	gain	17	17p11.2	16,475,319	20,564,580	4,089,261	Hoyer, 2007	MR, short stature
103	gain	17	17p11.2p11.2	16,557,249	20,289,720	3,732,471	Koolen et al. 2008	MR, hypotonia, microcephaly, abnormal thumbs
104	gain	17	17p11.2	16,622,658	20,325,270	3,702,612	Decipher database	No clinical data available
105	gain	17	17p11.2p11.2	16,763,408	20,162,287	3,398,879	Aradhya, 2007	MR, absent speech
106	loss	17	17p11.2	16,929,805	20,671,185	3,741,381	Rosenberg et al. 2006	MR, behavioural problems, FD
107	loss	17	17q11.2	26,178,327	27,177,418	999,091	Decipher database	No clinical data available
108	loss	17	17q11.2-q11.2	26,916,322	28,096,064	1,179,742	Menten et al. 2006	MR, microcephaly, long eyelashes, long columella, deep presacral groove, lacrimal duct stenosis
109	loss	17	17q21.31	40,523,389	41,520,916	997,527	Hoyer, 2007	MR, ventricle septum defect, short stature, seizures
110	loss	17	17q21.31	40,939,751	42,065,981	1,126,230	Shaw Smith et al. 2004	Severe MR, short stature, mild contractures, patchy skin pigmentation
111	loss	17	17q21.31	40,939,751	42,065,982	1,126,231	Krepischi-	MR, FD, seizures,

							Santos 2006	brachycephaly, occipital groove, hypopigmented skin, atrial septal and ventricular septal defects, and persistent ductus arteriosus
112	loss	17	17q21.31 q21.31	41,070,957	41,581,826	510,869	Koolen et al. 2008	MR, hypotonia, FD, ventriculomegaly
113	loss	17	17q21.31 q21.31	41,073,486	41,515,681	442,195	Aradhya, 2007	MR, growth retardation, seizures
114	gain	17	17q21.33	45,093,545	46,196,038	1,102,493	Friedman et al. 2006	mild MR, FD, microcephaly, conductive hearing loss
115	loss	17	17q23.2q 24.1	60,777,756	62,676,690	1,898,935	Menten et al. 2006	MR, psychiatric disorder, macrocephaly
116*	gain	17	17q25.1q 25.1	69,320,614	70,079,241	758,627	Koolen et al. 2008	Moderate MR, FD, growth retardation, kyphoscoliosis, small hands and feet with broad fingers/toes
117	loss	18	18q12.3-q12.3	39,644,137	41,812,640	2,168,503	Menten et al. 2006	MR, FD, short stature, growth retardation, narrow thorax, macrocephaly, prominent maxillary incisors
118	loss	18	18q21.2	50,552,638	51,742,365	1,189,727	Hoyer, 2007	MR, microcephaly, short stature, Hirschsprung disease, seizures, hypoplasia corpus callosum
119	loss	18	18q22.3-qter	67,465,993	76,439,842	8,973,849	Rosenberg et al. 2006	MR, short stature, hearing loss, mild FD, total anomalous pulmonary venous return, narrow and long fingers
120	loss	19	19p13.12 p13.2	10,291,150	13,749,674	3,458,524	Fan, 2007	severe MR, hypotonia, undescended testis, scoliosis, dextrocardia, hydronephrosis, macular hypoplasia
121	loss	19	19p13.11 p12	18,643,377	23,846,921	5,203,544	Hoyer, 2007	MR, macrocephaly, tetralogy of Fallot
122	loss	19	19q13.11	38,726,664	39,927,144	1,200,480	Shaw Smith et al. 2004	MR, growth retardation (prenatal), cutis aplasia
123	loss	19	19q13.11 q13.12	39465806	41915346	2449540	Koolen et al. 2008	MR, prenatal growth retardation, microcephaly, FD, dystrophic nails, hypotonia, delayed myelinisation
124	gain	19	19q13.11 q13.12	39,870,683	42,606,994	2,736,311	Hoyer, 2007	MR, macrocephaly, tetralogy of Fallot
125	loss	19	19q13.42 q13.42	60,626,627	63,008,986	2,382,359	Koolen et al. 2008	MR, FD, prominent forehead, abnormal ears with narrow auditary canals
126	gain	20	20q13.13 -q13.2	48,386,054	50,047,479	1,661,425	Menten et al. 2006	MR, FD, joint laxity, scoliosis, hyperelastic skin, webbed neck
127	loss	21	21q21.3q 22.12	28,802,339	34,984,201	6,181,862	Fan, 2007	MR, failure to thrive, agenesis corpus callosum, colpocephaly, white matter loss, absence septum pellucidum, nerve VI palsy,

								nystagmus, pectus, distal hypotonia.
128	loss	21	21q22.11 q22.12	32,329,260	35,362,570	3,033,310	Hoyer, 2007	MR, short stature
129	loss	21	21q22	37,497,428	43,098,299	5,600,871	Shaw Smith et al. 2004	MR, seizures, microcephaly, constipation, brachydactyly, syndactyly
130	gain	21	21q22.3q 22.3	45,480,127	45,756,958	276,831	Aradhya, 2007	MR, FD, vermian hypoplasia
131	gain	22	22q11	17,006,214	19,560,849	2,554,635	Decipher database	No clinical data available
132	loss	22	22q11	17,006,214	20,646,428	3,640,215	Decipher database	No clinical data available
133	gain	22	22q11.2-22q11.2	17,011,179	20,081,782	3,070,603	Miyake 2006	MR, brachydactyly, right cryptorchidism
134	loss	22	22q11.21	17,193,126	20,177,879	2,984,753	Krepischi-Santos 2006	MR, FD, common truncus arteriosus, multicystic left kidney and thymus agenesis.
135	loss	22	22q11.21	17,193,126	20,177,879	2,984,753	Krepischi-Santos 2006	MR, FD, hypothyroidism, microcephaly, slight aortic arch ectasy, upper vaginal agenesis, very rudimentary uterus, right kidney agenesis
136	loss	22	22q11.21	17,193,126	20,177,879	2,984,753	Krepischi-Santos 2006	MR, FD, schizophrenia
137	loss	22	22q11.21 q11.21	17,374,665	19,970,270	2,595,605	Koolen et al. 2008	MR, FD, growth retardation,
138	loss	22	22q11.21 q11.21	17,374,665	19,970,270	2,595,605	Koolen et al. 2008	MR, FD, bilateral postaxial polydactyly
139	loss	22	22q11.21 q11.21	17,374,665	20,124,628	2,749,963	de Vries et al. 2005	MR, FD
140*	gain	22	22q11.21 q11.21	18,984,318	20,187,133	1,202,815	Koolen et al. 2008	Severe MR, cataract, autism
141	loss	22	22q12.1	26,138,764	27,552,525	1,413,761	Friedman et al. 2006	MR, microcephaly, hypertrichosis and prominent brows, bifid uvula, undescended testis
142	loss	22	22q12.2q 12.2	27,394,025	28,959,779	1,565,754	Menten et al. 2006	MR, myopia, nasal speech, cleft uvula, pulmonary stenosis
143	loss	22	22q13.33 -q13.33	47,682,787	49,896,742	2,213,955	Menten et al. 2006	MR, hypotonia, adduction of thumbs, claw toes, syndactyly 3-4 fingers, dorsiflexion of the wrists
144	loss	22	22q13	49,413,465	49,514,104	100,639	Decipher database	No clinical data available
145	gain	X	Xp11.22p 11.23	46,703,690	54,174,129	7,470,439	Fan, 2007	MR, FD, seizures, growth retardation, clinodactyly fifth fingers, single transverse crease right hand, small hands and feet, hypotonia, autism
146	gain	X	Xq13.2q2 1.1	72,255,739	79,274,093	7,018,354	Koolen et al. 2008	MR, FD, growth retardation, broad thorax, cryptorchidism
147	loss	X	Xq22.2q2	102,418,89	103,075,340	656,450	Aradhya,	MR, hypotonic cerebral

			2.3mat	0			2007	palsy, delayed myelinisation in brain and thin corpus callosum
148	gain	X	Xq22.3	103,026,395	107,388,111	4,361,716	Krepischi-Santos 2006	MR, FD, hypotonia, microcephaly, trigonocephaly, hypospadias
150	gain	X	Xq25q26	128,430,890	133,134,713	4,703,823	Koolen et al. 2008	mild MR, FD, growth retardation (pre- and postnatal), microcephaly, cleft palate, kyphoscoliosis, hypospadias and cryptorchidism, short fingers and toes, clinodactyly
151	loss	X	Xq26.3q27.3mat	137,029,426	141,906,258	4,876,832	Aradhya, 2007	MR, macrocephaly, factor IX deficiency
152	loss	X	Xq27	139,618,609	140,661,097	1,042,488	Krepischi-Santos 2006	MR, bilateral coronal craniosynostosis
153	gain	X	Xq28	152,526,890	152,904,222	377,332	Decipher database	No clinical data available
154	gain	X	Xq28	153,272,076	154,451,934	1,179,858	Krepischi-Santos 2006	MR, dolichocephaly, obesity, hypogenitalism

(*Parent(s) unavailable thus validation of *de novo* status of CNV not possible)
