# Supplementary material for: Forging Links between Human Mental Retardation–Associated CNVs and Mouse Gene Knockout Models
Source: PLoS Genet. 2009 Jun 26;5(6):e1000531. doi: 10.1371/journal.pgen.1000531 (PMC2694283; doi:10.1371/journal.pgen.1000531)
Supplement: Table S3 — Matching patients' secondary clinical features to MGI mouse phenotype categories. For each set of CNVs grouped by secondary clinical features, the MGI phenotypic categories tested against are shown with an ‘X’. As CNVs grouped by secondary clinical features are subsets of the entire set of MR–associated CNVs, we sought to limit the number of statistical tests performed by considering only a subset of all MGI phenotypic terms. Thus, one of us (BVD) selected the most relevant categories (from a total of 33) of MGI phenotypic terms that only then were tested for significant enrichments. (0.13 MB PDF) [file pgen.1000531.s005.pdf]

	Human secondary clinical features groups						
**MGI Phenotypic section**	Brain abnormality	Cleft Palate	Eye abnormality	Facial abnormality	Heart abnormality	Seizures	Urogenital abnormality
MP:0001186 pigmentation			X				
MP:0002006 tumorigenesis							
MP:0002873 normal							
MP:0003012 no analysis							
MP:0003631 nervous system	X		X			X	
MP:0005367 renal/urinary system							X
MP:0005369 muscle					X		
MP:0005370 liver/biliary system							
MP:0005371 limbs/digits/tail							
MP:0005372 life span-post-weaning/aging							
MP:0005373 lethality-postnatal							
MP:0005374 lethality-prenatal/perinatal							
MP:0005375 adipose tissue							
MP:0005376 homeostasis/metabolism							
MP:0005377 hearing/vestibular/ear	X						
MP:0005378 growth/size							
MP:0005379 endocrine/exocrine gland							
MP:0005380 embryogenesis							
MP:0005381 digestive/alimentary							
MP:0005382 craniofacial	X	X		X			
MP:0005384 cellular							
MP:0005385 cardiovascular system					X		
MP:0005386 behaviour/neurological	X		X			X	
MP:0005387 immune system							
MP:0005388 respiratory system							
MP:0005389 reproductive system							X
MP:0005390 skeleton							
MP:0005391 vision/eye	X		X				
MP:0005392 touch/vibrissae							
MP:0005393 skin/coat/nails							
MP:0005394 taste/olfaction	X						
MP:0005395 other phenotype							
MP:0005397 hematopoietic system							
